# Two *Human papillomavirus 11* complete genomes recovered from inverted sinonasal papillomas in humans

**DOI:** 10.1128/mra.01184-23

**Published:** 2024-03-05

**Authors:** Sarah Bouzidi, Julien Puech, Marta Fulla, Xavier González-Compta, Hélène Pere, Laia Alemany, David Veyer, Ignacio G. Bravo

**Affiliations:** 1 Laboratory MIVEGEC (Univ Montpellier CNRS, IRD), French National Center for Scientific Research (CNRS), Montpellier, France; 2 Laboratoire de Virologie, Service de Microbiologie, hôpital européen Georges Pompidou, Assistance Publique-Hôpitaux de Paris, Paris, France; 3 Unité de Génomique Fonctionnelle des Tumeurs Solides, Centre de Recherche des Cordeliers, INSERM, Université Paris, Paris, France; 4 Otorhinolaryngology Department, Hospital Universitari de Bellvitge, L'Hospitalet de Llobregat, Spain; 5 Unit of Molecular Epidemiology and Genetics (UNIC EMG), Cancer Epidemiology Research Program, Catalan Institute of Oncology, ICO, L'Hospitalet, Barcelona, Spain; 6 Bellvitge Biomedical Research Institute (IDIBELL), L'Hospitalet, Barcelona, Spain; 7 CIBER en Epidemiología y Salud Pública (CIBERESP), Madrid, Spain; Rega Institute, Leuven, Belgium

**Keywords:** HPV, infection and cancer, inverted sinonasal papilloma, papillomavirus, HPV11

## Abstract

We communicate here two complete *Human papillomavirus 11* (HPV11) genomes recovered from one transitional and from one squamous inverted sinonasal papilloma, a rare proliferative disease in humans. Both genomes belong to the HPV11_A2 sublineage.

## ANNOUNCEMENT

Inverted sinonasal papillomas (ISP) are rare proliferative diseases of the nasal fossae and of the ethmoidal and maxillary sinuses, commonly associated to occupational exposures to organic solvents and to chronic inflammation (1). The role of Human papillomaviruses (HPVs) as etiological agents of ISP and the interpretation of HPV detection for the clinical prognosis of the disease are not well characterized yet (2, 3). In a large clinical study (4), only 2 out 46 ISP tested positive for HPVs. Here, we have characterized the complete viral genome component in these two ISP samples by high-throughput sequencing.

Protocols and use of samples were approved by the ethics committee of the Catalan Institute of Oncology (4). One sample originated from a woman living in Spain, diagnosed in 2009 of a transitional inverted sinonasal carcinoma, and the other sample originated from a man living in Spain, diagnosed in 2011 of a squamous inverted sinonasal carcinoma. Total DNA was extracted from formalin-fixed, paraffin-embedded samples using Maxwell automated commercial column extraction methods (4). Papillomavirus genomic material present in the extracted DNA was captured using over 22,000 probes, with an average length of 75 nucleotides, covering all HPV genotypes identified by the International Papillomavirus Reference Center, as described (5), using the KAPA Hypercapture and HyperExplore MAX reagents. Libraries were built using KAPA EvoPlus and dual-indexing reagents and sequenced on an Illumina MiSeq system (150 bp paired-end reads). Quality trimming and adapter clipping were performed on the raw reads using Trimmomatic v0.38 (6). Viral full genome sequences were assembled and annotated with breseq v0.38.1 (7) by aligning viable reads against the circular HPV11 GenBank M14119 reference genome, incorporating polymorphic sites, including indels, detected at a frequency above 0.5. Location of genomic features was performed by alignment against the annotated reference genome. For phylogenetic analysis, we retrieved the HPV11 full-genome GenBank entries used as reference to define the different variant lineages (8), aligned them using MAFFT v7.505 (9), and calculated a reference tree at the nucleotide level with RAxML-NG v1.1.0 (10) under the GTR + G + I evolutionary model. We added the novel sequences to the pre-computed alignment using MAFFT v7.505 (9) and applied the evolutionary placement algorithm of RAxML v8.2.12 (11) to place the novel sequences in the pre-computed HPV11 reference tree. The genetic distance was evaluated with the *ape* R package (12), using the TN93 substitution model.

The annotated HPV11 genomes are both 7,933 bp and have a G + C content of 41.1%. The viral genomes were complete and circular, with an average read depth of 4,216 and 6,987. They contained the complete *E6*, *E7 E1 E2*, *E5_γ*, *E5_δ*, *L2*, and *L1* ORFs, as well as the complete upstream regulatory region. The two viral genomes were identical in the *L1* ORF and differed in five positions across the full genome, corresponding to a divergence of 6.3 × 10^−4^ substitutions per site. Both sequences were unequivocally (posterior probabilities 0.9982 and 0.9993) assigned to HPV11 lineage A, sub-lineage A2, which is the most common HPV11 lineage (8).

## Data Availability

The HPV11 genomes herein communicated are available as GenBank entries OR669303 and OR669304 as biosamples SAMN38222584 and SAMN38222583, respectively, within the bioprojects PRJNA1039588 and PRJNA1039588, respectively. The corresponding raw reads are available as SRR26801485 and SRR26801484, respectively.
